# “Without the need for a second visit” initiative improves patient satisfaction with updated services of outpatient clinics in China

**DOI:** 10.1186/s12913-021-06260-3

**Published:** 2021-03-23

**Authors:** Jian Shen, Jun Zhang, Qiang He, Haihui Pan, Zhiqiang Wu, Liangming Nie, Hongfang Zhang, Shuning Liu, Yan Sun, Yaoqiang Du, Dongsheng Huang

**Affiliations:** 1 Department of Medical Administration, Zhejiang Provincial People’s Hospital, People’s Hospital of Hangzhou Medical College, No.158 Shangtang Road, Zhejiang, Hangzhou China; 2 Department of Blood Transfusion, Zhejiang Provincial People’s Hospital, People’s Hospital of Hangzhou Medical College, No.158 Shangtang Road, Zhejiang, Hangzhou China

**Keywords:** Without the need for a second visit (WNASV), Outpatient service, Patient satisfaction

## Abstract

**Background:**

To implement the “without the need for a second visit” (WNASV) initiative in our hospital by optimizing the outpatient clinic services via an upgraded information system, in order to increase the quality of outpatient medical services and improve patients’ satisfaction.

**Methods:**

An Internet-based care delivery approach was developed and applied to improve the delivery of health care services, simplify the treatment process, and reduce patient waiting time. The patient waiting time and consultation time in the outpatient clinics of our hospital during the peak service intervals and the proportions of various payment methods for outpatient services during the period from May 2017 to September 2019 were retrospectively analyzed. Also, the patients’ satisfaction with the outpatient process was surveyed.

**Results:**

The waiting time for consultation was shortened from 32.25 min to 28.42 min; the consultation time was shortened from 6.52 min to 3.15 min; and the waiting time for payment decreased from 7.40 min to 4.31 min. The proportion of payment via a counter was reduced from 86.80 to 21.79%, the proportion of self-service payment increased from 9.99 to 16.05%, and the proportion of payment during a consultation increased from 3.21 to 61.91%. The scores of the patients’ satisfaction with the outpatient services increased from an average of 89.10 points in 2017 to an average of 90.26 points in 2019.

**Conclusion:**

The continuous improvement of the service process markedly increases the efficiency of the outpatient services, and effectively improves patient’s satisfaction with the outpatient process, this initiative thus deserves further application.

**Supplementary Information:**

The online version contains supplementary material available at 10.1186/s12913-021-06260-3.

## Background

The “Without the need for a second visit” (WNASV) initiative was an important measure for reforming the review and approval systems in Zhejiang Province in 2017, and reflects the province’s determination in implementing people-centered development as proposed by Chinese President Jinping Xi [1]. Starting from the areas and issues that are most closely related to the lives of the general public and the business of enterprises, it makes full use of “Internet Plus government services” and big data to comprehensively promote good governance. In the spring of 2018, WNASV was officially seen in the Government Work Report delivered by Chinese Premier Keqiang Li, marking the national recognition of this practice.

Health care is closely related to people’s lives and attracts high social attention. The accessibility and affordability of medical services have long been problems for most patients and their families. In 2018, the People’s Government of Zhejiang Province urged the implementation of the WNASV initiative in health care services in Zhejiang Province, marking the extension of this initiative from government affairs to public services. In May 2018, Zhejiang Provincial Health Commission released Detailed Rules for Improving Health Care Services in 2018 [2], launching 10 major initiatives such as fewer queues for medical treatment, easier payment, and fewer errands for examinations. The weekly reporting system was established, along with the strengthened supervision, assessment, and whole-process monitoring mechanisms. Seventeen provincial hospitals and 155 municipal and county hospitals in Zhejiang Province were included in the monitoring mechanism. These 10 initiatives were further divided into over 40 items, and the Department of Medical Affairs of Zhejiang Provincial Health Commission ranked the performance of these hospitals every 15 days. Based on the data in the weekly reports, five items of particular concern including “online registration”, “queuing for medical treatment”, “intelligent payment system in outpatient clinics”, “intelligent payment system in wards”, and “reservation for examinations” were scored with “hearts” and released to the public through the media. Feedback from patient satisfaction surveys was used as an established yardstick for healthcare reform to promote the transformation and upgrading of medical services and health administration. In 2019, Zhejiang Provincial Health Commission further released “Without the Need for A Second Visit” reform for Improving Health Care Services in 2019 [3], deepen launching new 10 major initiatives to accelerate the transformation and upgrading of medical services and health services. The top 10 major initiatives of “Without the Need for A Second Visit” reform of the field of medical and health services in 2018 and 2019 are listed in Table 1.

**Table 1:** “Without the need for a second visit” reform: top 10 major initiatives of medical and health services

2018	2019	Continuous improvement
fewer queues for medical treatment	More reassuring to give medical advice at Primary hospital	Deepening reform
Easier payment	Payment after medical treatment and payment by credit	Deepening reform
fewer errands for examinations	Easier inspection	Deepening reform
Convenient hospitalization	More convenient to brush your face for medical treatment	Deepening reform
More considerate service for the convenience of the people	One step of medical service	Deepening reform
Faster first aid	/	/
More convenient dispensing	All in one health service card	Deepening reform
More warm mother and child health services	Integration of birth services	Deepening reform
Easier referral	/	/
Develop ‘Internet +’ health	Richer for “Internet + “health	Deepening reform
/	No need to run for blood service	New reform
/	More transparent to give vaccination	New reform

Patients’ satisfaction with outpatient services is a key indicator for evaluating the service capacity of a medical institution. Many studies have shown that a variety of factors in outpatient clinics are significantly correlated with outpatient satisfaction [4–6]. Therefore, how to properly address the problems of multiple long queues for medical treatment and the low efficiency of payment is particularly important for completing the entire treatment process. These concerns are also the priority of the WNASV initiative, the most notable of which are the implementation, evaluation, and ranking of smart outpatient services to help to shorten waiting times [7]. Therefore, the aim of current study was to summarize the implementation of the WNASV initiative in Zhejiang’s largest tertiary hospital under the direct leadership of the Zhejiang Provincial Health Commission. We focused on the hospital’s efforts in delivering smart outpatient services in terms of optimized treatment process, improved treatment services, and reformed treatment modes. In this longitudinal research, we quantified the changes in patient visit time and payment modes before and after the adoption of the initiative and their impacts on patient satisfaction [8].

### Optimized outpatient service process

**Fig. 1:**
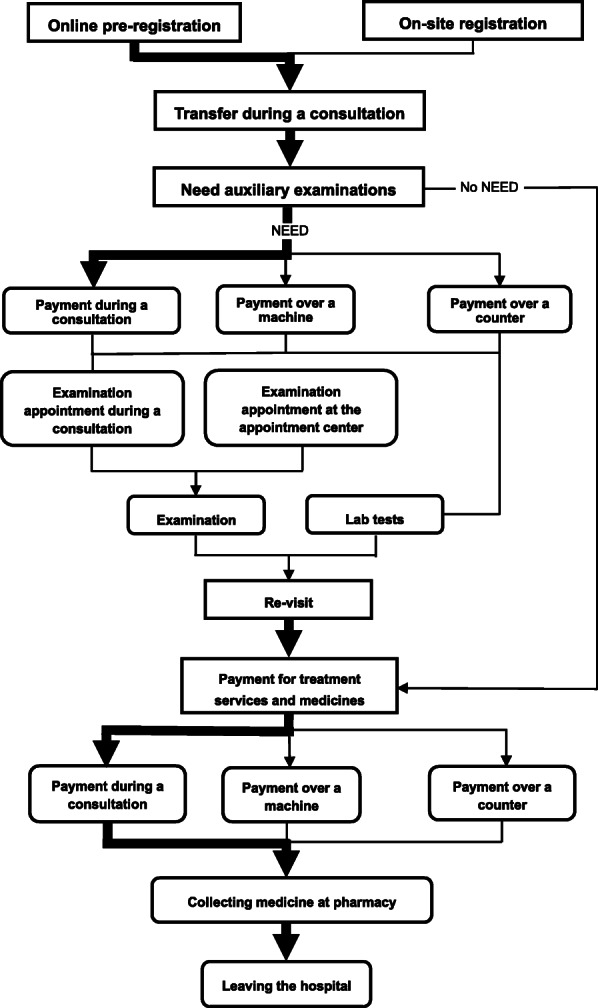
Flow chart of outpatient process in the pilot hospital. The thickness of lines and arrows is inversely proportional to patient waiting time

Before the adoption of the WNASV initiative, the outpatients had to be queued five or six times for registration, consultation, examinations, tests, payment, and collecting prescription from a pharmacy; also, they had to pay three times for registration, auxiliary examinations, and medicine. Occasionally, outpatients also had to be queued for payment at extra cost in front of a counter, which was highly time-consuming [9]. Since much time was spent in non-consultation affairs, the treatment efficiency was poor, and the patient’s satisfaction was low. Since the adoption of smart outpatient services in the pilot hospital in June 2018, the software and hardware facilities for smart payment were introduced and the cooperation with online payment services was strengthened; thus, the adequate integration of “Internet Plus” technology with the outpatient process (except for consultations) dramatically improved the registration and payment modes [10]. Meanwhile, with the gradual implementation of the WNASV items including “online pre-registration” and “examination appointment”, the pilot hospital further improved the outpatient process by reducing both the time of patients took to return to the hospital for further examination and treatment and the time at the hospital spent in line. Flow chart of outpatient process in the pilot hospital is illustrated in Fig. 1.

### Optimized outpatient payment process

In the smart outpatient payment system of the WNASV initiative, the outpatient clinics should offer at least three payment modes: self-service payment, payment during a consultation, and payment via a mobile terminal [11]. The payment system must have multiple functions including patient identification, fee settlement, mobile payment, and medical insurance claims; it should enable offline and online real-name authentication and be able to identify at least five ID cards/documents including the ID Card, social security card, resident electronic health card, hukou book, and passport; and it should support at least six payment pathways including cash, check, bank cards, WeChat, Alipay, and social security cards. It was required that the introduction of the WNASV initiative should be completed in all the included provincial and municipal hospitals by the end of June 2018.

Payment during consultation was adopted in our hospital in 2017. However, it was applicable only to those patients holding a Hangzhou citizen card and to those with a Zhejiang Provincial medical insurance card; furthermore, the fees out of the patients’ own pockets could only be paid via a pre-charge mode in the smart medicine services. As a result, the payment during a consultation had not been fully implemented. In June 2018, in response to the WNASV initiative, the hospital introduced the Alipay and WeChat Pay through in-depth cooperation with Internet companies such as Alibaba. Meanwhile, the updated software and hardware facilities have continuously improved the “payment during a consultation” in the hospital information system (HIS). With the use of small self-service devices, the out-of-pocket expenses are conveniently paid via Alipay or WeChat Pay within seconds. On the other hand, measures are taken to promote payment to occur during a consultation. Video-based training and IT engineer instruction at the doctor’s office ensure that each doctor is familiar with the payment steps, so that the patients can complete their payment smoothly. After a patient finishes his/her diagnosis and treatment in the doctor’s workstation, the system will automatically enter the payment interface; this way, payment is more intimately integrated into the diagnosis and treatment process, and the proportion of payments occurring during a consultation is increased.

Self-service payment includes bank card payment, Alipay, and WeChat Pay, and is completed by QR code scanning under the guidance of hospital staff. The finance department, the temporary engineering department, the information center, the volunteer service center, and many other relevant departments established a self-service payment WeChat group. Any problems found during the services are submitted in timely fashion to the WeChat group, and the relevant department will offer solutions online, which effectively increases the adoption of self-service payment. During the implementation of self-service payment, the information center continuously records the patient experience, upgrades the software program, and thus improves the overall service experience; also, when there are long queues at the counters, the self-service guides actively instruct the patients to pay their fees over the self-service machines, thus alleviating the stress of queuing for registration and payment in outpatient clinics.

The counter is more suited for patients who pay in cash, remain unfamiliar with mobile terminal payment, and/or have doubts about social security or expenses. In response to the requirements of the WNASV initiative, we integrated the counters by introducing Alipay and WeChat Pay to make payments via QR Codes and mobile phones. The mobile terminal payment method is used to replace cash transactions, so as to speed up the payment at counters, reduce the charging errors, and increase the accuracy of payment [12]. The hospital also launched a self-service invoice printer, which is managed by a staff member from the toll collection office to help the patient receive an invoice without queuing again in front of another counter. The service center in the outpatient hall offers financial information service and medical insurance service. The full-time staff members handle clerical tasks including correction of the name on invoices, refunds, and issuing a receipt or invoice, which improves the efficiency of the counters.

## Methods

### Design of the study protocol

The ethical approval and consent to participate were supported by the Ethics Committee for Zhejiang Provincial People’s Hospital in Hangzhou, Zhejiang, China. The hospital is the largest comprehensive public hospital in Zhejiang Province, and the composition of patients conforms to the average level of domestic patients. As a pilot institution, the hospital began to implement smart outpatient services in June 2018. By using a longitudinal research method, we analyzed the changes in patient waiting time and patient satisfaction before (from May 1, 2017 to May 31, 2017) and after the implementation of the reform (from November 1, 2018 to September 31, 2019). The period from June 2018 to October 2018 was excluded from the study period mainly due to the switching between major information systems and the Chinese National Day holiday. The subjects were all outpatients who sought medical treatment in the outpatient clinics of the pilot hospital from May 1, 2017 to September 31, 2019.

### Patients’ satisfaction

1. There is no widely accepted definition of patient satisfaction. According to different conceptual models, patient satisfaction can be defined as follows: 1) satisfaction with the health system as a whole; 2) satisfaction with the quality and type of medical and health services obtained; 3) satisfaction with non clinical aspects of medical and health services (waiting time, appointment, expense reimbursement, etc.); 4) satisfaction or recognition of policy changes [13] . And we agree that that Patients’ satisfaction with medical institutions refers to the evaluation of patients’ health, disease, quality of life and other requirements after comparing the medical services provided by medical institutions with their expectations [14].
2. Patient satisfaction is a subjective evaluation to improve the objective management factors of the hospital. Therefore, in the analysis of patient satisfaction, we need to directly associate the patient satisfaction with the objective management indicators of the hospital. As the related time involved in the article is the performance of the objective management indicators (the waiting time for consultation, the waiting time for medicineand so on).
3. Although the gender, age, family level and wealth status of patients are the factors influencing patient satisfaction and are also in the patient satisfaction evaluation system, these indicators are not involved in the optimization of hospital management. This paper does not do in-depth discussion, because in-depth discussion of these factors will lead to deviation from the original intention of optimizing hospital management.

### Data collection

All data of time were collected from the hospital information system (HIS) of the pilot hospital. One single outpatient number was assigned for each patient when he/she visited this hospital. This number was used for pre-appointment, registration, consultation, prescription, test/examination, payment, and medicine collection. Information including diagnosis, medical histories, and prescriptions corresponding to their outpatient number was recorded in the HIS.

The waiting time was divided into the waiting time for consultation and the waiting time for medicine collection. The waiting time for consultation was calculated according to the patient registration time and the time of the consultation: (waiting time for consultation) = (the login time of the outpatient number on the computer of the doctor) – (the time of the outpatient number recorded in the registration system). The waiting time for medicine collection was calculated based on the time of sending the prescriptions for a given outpatient number from the doctor’s computer and the time of leaving hospital: (waiting time for medicine collection) = (the time of displaying the outpatient number on the LED screen of the pharmacy) – (the time of sending the prescriptions of outpatient number from the doctor’s computer). We also counted the number of payments during a consultation, self-service payments, and payments over a counter in the pilot hospital every month; the data on the queuing time for payment over a counter during peak hours of weekly outpatient visits (9:00 am – 10:00 am on a Monday, according to the distribution of hospital outpatient visits) were also collected.

In the process of doctor’s outpatient service, after the last patient’s visit, the outpatient HIS will automatically remind the next patient in the waiting area to see a doctor. When the patient enters the clinic, the doctor enters the patient’s visit number in the outpatient HIS, and the reminder automatically ends. The doctor can use the patient’s visit number to view the patient’s previous visit records to improve the reception efficiency. Therefore, the waiting time is equal to the login time of the patient’s registration number on the computer of the attending doctor - the time when the patient’s medical registration is entered in the registration system; after the patient’s outpatient visit is completed, the outpatient HIS is the prescription corresponding to the patient’s visit number is automatically sent to the outpatient pharmacy information system. The pharmacy of our hospital has automatically distributed the medicine in the whole process. After the outpatient medicine is prepared, the patient will be reminded to take the medicine on the LED screen of the outpatient pharmacy, so the waiting time of the patient’s outpatient drug taking = the real time of the patient’s visit number on the LED screen of the drug room - the corresponding prescription sending time of the patient’s visit number.

### Survey on patient satisfaction

The patient’s satisfaction with the outpatient service process was regularly monitored on a monthly basis during the study period, upon the approval of the Hospital Ethics Committee. The questionnaire was designed according to the patient’s procedure of seeking medical treatment in our hospital (Supplementary Table 1). The questionnaire includes three aspects: 1) the type of patients seeking medical treatment, including whether they are newly diagnosed patients, the source of patients, the type of expenses and the specialty of visiting doctors; 2) the satisfaction degree of each link of the visit, including the attitude of the staff, the detailed degree of interrogation and physical examination, the degree of respect for the privacy of patients, the overall service attitude of doctors, and the overall service status of nurses;3) satisfaction degree of waiting time in each link of this visit, including waiting time. All items with a 5-Point Likert scale were scored as 5, 4, 3, 2, or 1 point if answered with strongly agree, agree, neither agree nor disagree, disagree, or strongly disagree, respectively. The satisfaction was calculated by using the following formula: (frequency of answer “strongly agree” × 5 + frequency of answer “agree” × 4 + frequency of answer “neither agree nor disagree” × 3 + frequency of answer “disagree” × 2 + frequency of answer “strongly disagree” × 1) / (number of people surveyed × 5). “The number of people surveyed × 5” means that each patient has five degree of satisfaction: strongly agree, agree, neither agree nor disagree, disagree, or strongly disagree. The effective rate of the questionnaire was 100%. The survey objects are required to meet the following conditions: 1) outpatients over 18 years old; 2) the patients does not include department of psychiatry; 3) the informants explained the informed consent to the patients, and only those who agreed to attend the surgery were included and asked to fill in the satisfaction questionnaire. The sample hospitals are the participating units of China’s Requirements for third-party evaluation of patient satisfaction in medical institutions (the only group standard of patient satisfaction in China, NO:T/ZYYX 001–2020). A total 157 of hospitals participated in the construction of China’s patient satisfaction evaluation system (Group standard), and gained a common understanding of China’s patient satisfaction evaluation in China.

### Statistical analysis

Statistical analysis was performed by using SPSS 18.0 software package. The measurement data are expressed as $\overline{\mathrm{x}}\pm \mathrm{SD}$, and the comparisons of the measurement data were based on logistic regression model for exploring the influencing factors of patients’ satisfaction. The *p*-value of < 0.05 was considered statistical significance. We assumed that the period from May 2017 to May 2018 was the period during which the routine operation data of the outpatient clinics were obtained when the outpatient system A was still running before the adoption of the WNASV initiative, and the period from November 2018 to September 2019 was the period during which the outpatient operation data were obtained after the optimized outpatient system B was adopted after the adoption of the WNASV initiative. The period from June 2018 to September 2018 was regarded as a buffer period for the optimization of informatization, during which system B was applied and the outpatient operation data were also synchronized to the system B database. Each system separately recorded the time at all links of outpatient services, calculated the coefficients of variation due to the change of information systems, and thus enabled homogeneity correction for the time recorded in the two systems.

## Results

### Changes in waiting time during the peak interval before and after continuous improvement by the WNASV initiative

**Fig. 2:**
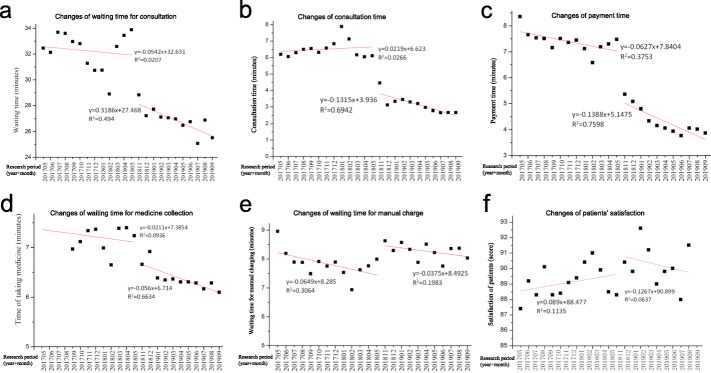
The changes in the waiting time and patient satisfaction before and after continuous improvement. **a** Level and trend Change of waiting time for consultation before and after improvement; stand for waiting time for consultation, the trend and data has declined significantly before and after improvement. **b** Level and trend Change of consultation time before and after improvement; stand for consultation time, the trend and data has declined significantly before and after improvement. **c** Level and trend Change of payment time before and after improvement; stand for payment time, the trend and data has declined significantly before and after improvement. **d** Level and trend Change of medicine collection time before and after improvement; stand for medicine collection time, the trend and data has declined significantly before and after improvement. **e** Level and trend Change of waiting time for manual charge before and after improvement; stand for medicine collection time, the data has increased significantly before and after improvement. **f** Level and trend Change of the patients’ satisfaction with the outpatient process before and after continuous improvement. The data has increased significantly before and after improvement

The changes in the waiting time during the peak interval before and after continuous improvement in the two research periods are summarized in Table 2. The waiting time for consultation was shortened from 32.25 min to 28.42 min (Fig. 2a); the consultation time was shortened from 6.52 min to 3.15 min (Fig. 2b); the payment time was shortened from 7.40 min to 4.31 min (Fig. 2c); the waiting time for medicine collection was shortened from 7.24 min to 6.35 min (Fig. 2d); the waiting time in front of a counter increased from 7.83 min to 8.27 min (Fig. 2e).

**Table 2:** Changes in waiting time during peak interval before and after continuous improvement

Study phase	201,705–201,805	201,811–201,909	T-value	*p*-value
Average sample size (Times)	83,046 ± 5744	93,703 ± 9071	/	/
Waiting time for consultation (minutes)($\overline{\mathrm{x}}\pm \mathrm{s}$)	32.25 ± 1.46	28.42 ± 3.51	12.928	**0.002**
Consultation time (minutes)($\overline{\mathrm{x}}\pm \mathrm{s}$)	6.52 ± 0.52	3.15 ± 0.52	247.503	**< 0.001**
Payment time (minutes)($\overline{\mathrm{x}}\pm \mathrm{s}$)	7.40 ± 0.40	4.31 ± 0.53	266.014	**< 0.001**
Waiting time for medicine collection (minutes)($\overline{\mathrm{x}}\pm \mathrm{s}$)	7.24 ± 0.27	6.35 ± 0.22	72.418	**< 0.001**
Waiting time for manual charge (minutes)($\overline{\mathrm{x}}\pm \mathrm{s}$)	7.83 ± 0.46	8.27 ± 0.28	7.607	**0.011**
Degree of satisfaction(scores)($\overline{\mathrm{x}}\pm \mathrm{s}$)	89.10 ± 1.03	90.26 ± 1.37	5.109	**0.035**

Bold signifies statistically significant coefficient between “201,705–201,805” and “201,811–201,909” (*p* < 0.05)

### Changes in the proportions of different payment modes before and after continuous improvement

**Fig. 3:**
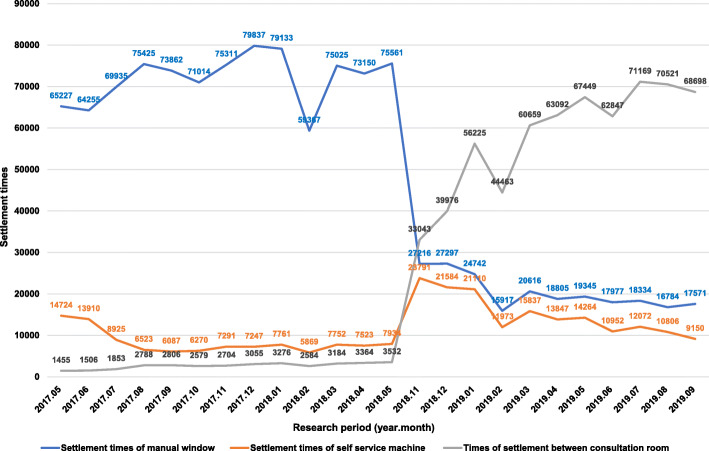
Changes in the proportion of payments during a consultation before and after continuous improvement

As shown in Fig. 3, changes in monthly settlements and proportion of payments before and after continuous improvement are illustrated. The total number of payments was 1,107,604 from May 2017 to May 2018, and the number of payments over a counter was 937,102; the proportions of payment during a consultation, self-service payment, and payment over a counter were 3.21%, 9,99, and 86.80%, respectively. After continuous improvement, the number of payments during a consultation gradually increased. A total of 1,106,885 payments occurred during the period from November 2018 to September 2018, among which the number of payments over a counter was 252,699; the proportions of payments during a consultation, self-service payment, and payment over a counter were 61.91%, 16,05, and 21.79%, respectively. Comparisons of the proportions of these payment modes between two specific periods (2017/05/01–2018/05/31 vs 2018/11/01–2019/09/31) showed that the proportion of payments during a consultation remarkably increased whereas the proportion of payments over a counter dramatically declined, and the differences were statistically significant (both *p* < 0.01).

### Change in the patients’ satisfaction with the outpatient process before and after continuous improvement

Before the continuous improvement (from May 2017 to May 2018), the average patient’s satisfaction score on the outpatient process was 89.10. After the continuous improvement (from November 2018 to September 2019), the average patient’s satisfaction score on the outpatient process significantly increased to 90.26 (Fig. 2f).

## Discussion

As demonstrated in our current study, the optimized outpatient process after the adoption of smart outpatient services in the WNASV initiative has markedly shortened the patient’s time in the health care process, changed the payment mode, and increased the patients’ satisfaction with outpatient services. An explanation of these changes follows below.

First of all, outpatients typically have to line up multiple times to access outpatient services, which is highly time-consuming and will inevitably lower their satisfaction with their experience. Long wait times are associated with low patient satisfaction scores [7–9]. After the smart outpatient services in the WNASV initiative were adopted in the pilot hospital, the software and hardware facilities were upgraded for smart medical treatment, which allowed the full integration of “Internet Plus” technology into all aspects of the outpatient process and reduced the steps and intervals of outpatient services including in registration, waiting for consultation, tests, examinations, payment, and medicine collection. Accordingly, the waiting time for consultation, consultation time, waiting time for payment, and waiting time for medicine collection substantially decreased. In addition, information technology was used to strengthen the role of the appointment mode in the outpatient process, which could accurately evaluate the timing of the appointment and give the patient a friendly reminder to visit the hospital on time. The adequate integration of the “Internet Plus” technology in the outpatient process reduced the number of revisits and shortened the long queues for medical treatment. In China, the average consultation time is much shorter than many other western countries. Thus, the implementation of these measures allowed the patients to spend adequate time communicating with their doctors, which is the core of the medical consultation process, and will substantially increase the patient’s satisfaction with the treatment process.

Secondly, payment is an indispensable part of the outpatient process and has long been a major concern in outpatient administration. Previous studies [10–12, 15] have shown that outpatients spend most of their time waiting and queuing. The original adoption of the pre-paid model in smart medicine required two extra steps (recharge and refund) after payment and did not shorten the queues in an outpatient hall [4]. Hospital informatization plays an important role in optimizing the outpatient process [5, 16]. To facilitate the implementation of the WNASV initiative in health care services, the pilot hospital introduced third-party payment terminals, so as to transform the advantage of Internet Plus technology in processing big data into another tool to ease the pressure on outpatient clinics. Mobile payment has high security standards in terms of network software and hardware configurations, payment platform and user terminal design, and the network security environment. Meanwhile, the system is operated in a closed environment and no toll collector is required, which can prevent errors caused by the manned operation and thus avoids unnecessary disputes between doctors and patients [6, 17]. Most outpatients are young and middle-aged individuals. It was found that 60.7% of people aged 50–70 years used smart phones [18], and the vast majority of patients aged 70 years or older are escorted by their children when visiting the outpatient departments. As shown in our current study, payment during a consultation by using Alipay or WeChat Pay had been routinely applied during outpatient services in the period from May 2017 to May 2018. After the optimization of informatization became stabilized in November 2018, the number of payments during a consultation increased continuously from 1455 in May 2017 to 33,043 in November 2018 and to 68,698 in September 2019. Payment during a consultation enables “zero errands” and “zero waiting”, which meets the requirements of most patients. Some experts found that non-cash transactions assisted by the use of third-party payment platforms could effectively address problems such as long waiting times for registration and payment, crowded waiting areas, and secondary infections during hospital visits, thus improving patient experience and promoting a shared understanding between doctors and patients [17, 19].

Thirdly, payment does not mean the end of the medical service but marks the beginning of another service. The patient will travel to other medical departments such as laboratories, examination rooms, and the pharmacy. The outpatient satisfaction score was 89.10 ± 1.03 during the period from May 2017 to May 2018; after the adoption of the smart outpatient services in the WNASV initiative, it was dramatically increased to 90.26 ± 1.37 during the period from November 2018 to September 2019. Analysis based on the satisfaction scale has shown that medical quality, service attitude, and time cost are the most concerning issues during an outpatient visit [20–23]. Hospital adopted the eliminate, combine, rearrange and simplify (ECRS) method to re-integrate and reorganize the various links and steps of the outpatient service process. Meanwhile, they vigorously strengthened informatization by upgrading the HIS, applying a large number of self-service systems, implementing full WIFI coverage, and releasing a mobile App for the hospital. As a result, the overall satisfaction score of outpatients has increased 23% per year by year [24, 25]. Although their measures differ from ours, both hospitals attached great importance to informatization, in particularly the application of Internet techniques, and achieved similar effectiveness. The satisfaction of outpatients plays an important role in improving the hospital’s social reputation and increasing economic income. *Zheng* et al surveyed the outpatients and concluded that, in addition to the attitudes and skills of the doctors, the attitudes of other medical staff also affected the overall satisfaction of the outpatients with the service process (*p* = 0.032) [11, 12, 26]. In our current study, thanks to efforts on multiple reforms, including services provided by self-service guides and counter staff, the patients’ satisfaction with the outpatient process was significantly improved.

Fourth: The consultation time is shortened from 6.52 min to 3.12 min in this study, the major concerns of which are as follows: (1) In this study, the shortening of consultation time is beyond our initial expectation, as the prolongation of consultation time is one of the factors of the increase of hospital service quality and patient’s satisfaction [27–30]. Meanwhile, as mentioned above, there is an increase of patient’s satisfaction. At first, we were also confused for this data. But after in-depth analysis, we found that this data and results reflect the actual situation of medical treatment in Chinese society. As for the tendency of medical treatment, Chinese patients prefer to visit Chinese public tertiary general hospital to going for further consultation or dispensing which makes the number of patients in Chinese public tertiary general hospital in relevant areas much larger than that in Grass-roots Hospitals and private hospitals. Although the Chinese government is committed to hierarchical diagnosis and treatment by learning from foreign medical treatment models, and strives to reduce the number of patients in Chinese public tertiary general hospital, the effect is not obvious for a short time. The number of outpatients in sample hospitals increased nearly 10,000 per month averagely after the reform. This can be explained by the increased preference of the sample hospital by the patients, resulting from the common assumption that the service is promoted after adopting the “WNASV” reform and optimizing the outpatient service process. (2) In the pilot hospital, the proportion of consultation time less than 3 min gradually increased after the reform according to the analysis, which is consisted by the patients going to the hospital either to acclaim the results of physical examination or obtain prescription dispensing for chronic diseases. This well demonstrated the wide acceptance of the underlining reform of the sample hospitals by the patients, including the process optimization from “Check for less running”, “More convenient dispensing”, “Easier inspection” and “One step of medical services” through the reform campaign called “WNASV” in 2018 and 2019.

Finally: For the research of patients’ satisfaction in many hospitals, they will set many factors to discuss patients’ satisfaction in the hospital, including the medical technology of the hospital, the location of the hospital, the price of the hospital and the cost performance of the hospital, which may not be included in this paper, but also the limitation of the research. But the author thinks that this often leads to the hospital patient satisfaction did not grasp the main factors that the hospital needs to solve, the function of the hospital is to protect the patient’s treatment, the convenience of the patient’s treatment is the embodiment of the patient’s actual satisfaction. Therefore, the limitation of this paper is also the specificity of this study on patient satisfaction.

An important difference between the questionnaire and many domestic questionnaires is that the questionnaire does not include the controversial dimensions. For example, the patient’s price, transportation and other factors are not completely controlled by the hospital itself. Therefore, the quality of medical technology is not suitable as the evaluation dimension of patient satisfaction, but more suitable to use objective indicators for evaluation. The questionnaire developed in this paper only retains the undisputed part to ensure the authenticity and credibility of the evaluation. Different patient characteristics, hospital characteristics, cultural environment characteristics will have an impact on the measurement results of patient satisfaction. Therefore, in the application of satisfaction results, the administrative departments should carefully consider the region, type and scale of different hospitals, considered the patient structure and individual characteristics of the hospital, eliminate the interference of these factors, so as to ensure the comparability and fairness in horizontal comparison.

### Limitations

1. An important difference between the questionnaire and many domestic questionnaires is that the questionnaire does not contain controversial dimensions. For example, the patient’s price, transportation and other factors are not completely controlled by the hospital itself; many articles on patient satisfaction include many factors, but many factors lead to the deviation of satisfaction results, which can not play a guiding role for the hospital.
2. Medical technology quality is not suitable as the evaluation dimension of patient satisfaction, but more suitable to use objective indicators for evaluation. The questionnaire prepared in this paper only retains the indisputable part to ensure the authenticity and credibility of the evaluation.
3. Different patient characteristics, hospital characteristics and cultural environment characteristics will affect the measurement results of patient satisfaction. But for the study of hospital patient satisfaction, we can not add all the factors into the analysis. Therefore, when applying the satisfaction results, the management department should carefully consider the region, type and scale of different hospitals, the patient structure and personal characteristics of hospitals, and eliminate the interference of these factors, so as to ensure the horizontal comparability and fairness comparison. We choose Zhejiang Provincial People’s Hospital as the sample hospital, which is relatively simple and limited. Only in this way can the research of patients’ satisfaction be more targeted.
4. The limitations of the study, the way of data acquisition and the degree of patient satisfaction in this study can not be achieved by every hospital, which leads to differences between our study and other hospitals.

## Conclusion

The wide application of Internet Plus techniques makes it easier for patients to accept new, smart outpatient services. While fully utilizing the opportunities and advantages of online payment technology, we also updated the on-site payment services in our hospital. Currently, our hospital is promoting the “face swiping for medical treatment” and “payment after hospital visit” services, which use face recognition technology and the personal credit system based on the ID card, citizen card, social security card, and/or bank card to allow patients to go through a smart hospital visit without bringing any cards. The brand-new hospital visit mode has also enacted the change of payment proportions. The adoption of new payment modes including the upgraded outpatient mobile terminals for self-service payment and the faster payment via counters, has reduced and shortened patient queues. The increased skills of toll collectors in the outpatient service center and the “green channel” for post-payment services has also offered humanized and efficient solutions. Safe and convenient payment methods and considerate services reassure the outpatients in our hospital. The online and offline O_2_O closed-loop service ecosystem in an intelligent hospital will improve hospital management efficiency and increase patient satisfaction. In summary, the continuous improvement of the service process markedly increases the efficiency of the outpatient services and effectively improves patient’s satisfaction with the outpatient process, and the WNASV initiative in health care services of Zhejiang Province thus deserves further application.

## Supplementary Information

**Additional file 1: Supplementary Table 1.** The questionnaire/survey used in this study.

## Data Availability

The datasets used and/or analyzed during the current study are available from the corresponding author or first author on reasonable request.

## Abbreviations

WNASV: Without the need for a second visit
HIS: Hospital information system
LED: Light Emitting Diode
WIFI: Wireless-Fidelity
ID: Identity document
O_2_O: Online to offline.
