# Supplementary material for: “Without the need for a second visit” initiative improves patient satisfaction with updated services of outpatient clinics in China
Source: BMC Health Serv Res. 2021 Mar 23;21:267. doi: 10.1186/s12913-021-06260-3 (PMC7986498; doi:10.1186/s12913-021-06260-3)
Supplement: Supplementary file 1 — Additional file 1: Supplementary Table 1. The questionnaire/survey used in this study. [file 12913_2021_6260_MOESM1_ESM.doc]

**Questionnaire of satisfaction and behavior style of outpatients in Zhejiang Provincial People's Hospital**

ID： Clinic Department： Phone Number：

**Dear Sir/Madam,**

Sincerely wish you keep health.

In order to continuously improve the quality of medical service and improve the level of medical service, our hospital will continue to carry out patient satisfaction and behavior survey activities. We sincerely invite you to participate in the activity, your valuable comments and suggestions are very important to us.

We sincerely appreciate your help and cooperation!

Thank you very much!

**A About the experience of registration**

1. What kind of registration do you use?

□Register the window □Appointment for registration (e.g. Phone, Internet, WeChat, etc.) □Self-service machine registration □The other approach

2. If you are a window registration, the queuing time is about ＿＿＿＿ minutes.

**B Service window communication**

3. Have you been to the following function window：

□Registration □Inspection center □Radiology department □Ultrasonic department □The electrocardiogram □Pharmacy

4. Did the window attendants treat you with respect?

□Very disrespectful □A little disrespectful □More respect □Very respectful

5. Did the staff at the window you visited listen to you carefully?

□Very carelessly □Not carefully □More carefully □Very carefully

**C About communication with your doctor**

6. Did the doctor treat you with respect?

□Very disrespectful □A little disrespectful □More respect □Very respectful

7. Did the doctor listen to you carefully?

□Very carelessly □Not carefully □More carefully □Very carefully

8. Did the doctor explain the problem in a way that you understood?

□Totally incomprehensible □barely understand □Basically understand □Fully understand

**D About communication with your nurses**

9. Did the nurse treat you with respect?

□Very disrespectful □A little disrespectful □More respect □Very respectful

10. Did the nurse listen to you carefully?

□Very carelessly □Not carefully □More carefully □Very carefully

11. Does the nurse explain the problem in a way that you can understand?

□Totally incomprehensible □barely understand □Basically understand □Fully understand

**E Environment and Identification**

12. Are road signs and instructions clear in the hospital?

□Very ambiguous □less clear □More clear □Very clear

13. What is your overall impression of the hospital facilities?(e.g. seats, elevators, drinking water equipment)

□Very dissatisfied □Less satisfied □satisfied □Very satisfied

14. Are hospital toilets clean and odor-free?

□Very unclean □less clean □More clean □Very clean

15. Is the spatial layout of the hospital convenient?

(such as the floor and distance of triage, registration, consulting room, examination and charge department)

□Very inconvenient □a little inconvenient□More convenient □Very convenient

**F Privacy protection**

16. Do the medical staff pay attention to protect your privacy during the treatment?

(e.g. whether the curtain is drawn during inspection)

□Very inattentive □Less attentive □More attention □Very attentive

**G Medical staff response**

17. Can your complaints and grievances be answered in a timely manner during the visit?

□Very untimely □Less timely □More timely □Very timely □I have no suggestions or complaints

**H Finally, please make an overall evaluation of this hospitalization**

18. Which of the following numbers best represents your overall assessment of the hospital?（1-10）

□1 The worst □2 □3 □4 □5 □6 □7 □8better □9 □10The best

**I If you wish, please provide written comments on hospital management and services below**

praise：

Suggestions and advice：

**J other questions**

19. Would you recommend this hospital to your relatives and friends?？

□Absolutely not □Basic won't □Maybe □Must be

20. Your gender： □Male □Female

21. Your age：

□under 20 years old □20 to 29 years old □30 to 39 years old □40 to 49 years old □50 to 59 years old □More than 60 years of age

22. Your highest education：

□Junior high school the following □High school or technical secondary school □Bachelor or junior college degree □Postgraduate

23. How to pay for your medical treatment：

□Out-of-pocket medical □The new rural cooperative medical care system □Urban medical insurance (employee/resident)□Free medical care

Thank you for your kind help. Please check again to ensure you have answered all the questions. Your feedback will help us to improve the service quality of patients.

Zhejiang Provincial People's Hospital, People’s Hospital of Hangzhou Medical College
